# Case report of seminal vesical schwannoma treated with conservative strategy

**DOI:** 10.1097/MD.0000000000022307

**Published:** 2020-09-18

**Authors:** Peng Zhang, Ming Yang

**Affiliations:** Department of Urology, Ningbo Medical Center Lihuili Hospital, Ningbo, Zhejiang, China.

**Keywords:** neurilemmoma, seminal vesicles

## Abstract

**Rationale:**

Schwannoma is a benign peripheral nerve sheath tumor composed of Schwann cells and caused by genetic mutation or deletion. It rarely occurs in seminal vesicles. The optimal therapic strategy for asymptomatic cases is still unclear.

**Patient concerns:**

A 42-year-old man presented no clinical symptoms. A mass in his left seminal vesicle was found incidentally in a computed tomography scan and transrectal ultrasound-guided biopsy revealed the mass was schwannoma.

**Diagnosis:**

The patient was diagnosed as schwannoma of the seminal vesicle with no significant extension to the surrounding tissues.

**Interventions:**

The patient underwent computed tomography or magnetic resonance imaging scans periodically to estimate the alteration of the lesion and further strategy.

**Outcomes:**

After 20-month follow-up, computed tomography scans showed no significant alteration to the lesion and no clinical symptoms were reported by the patient.

**Lessons:**

Conservative strategy might be an effective treatment option for asymptomatic patients with seminal vesical schwannoma. The period of follow-up depends on the size of the tumor.

## 1 Introduction

Primary neoplasms of seminal vesicles are extremely rare. Reported benign tumors include cystadenoma, papillary adenoma, leiomyoma, teratoma, neurilemoma, and epithelial stromal tumor.^[1]^ Primary malignant neoplasms include adenocarcinoma, leiomyosarcoma, angiosarcoma, carcinoid, seminoma, and cystosarcoma phyllodes.^[1]^

Schwannomas, also known as neurilemmomas, are caused by either mutations or deletions of both copies of the *NF2* gene on chromosome of 22q12.^[2]^ They are benign peripheral nerve tumors composed of well-differentiated Schwann cells,^[3]^ which are responsible for formation of the myelin sheath of peripheral nerve cells. As such, schwannomas may arise anywhere along the course of peripheral nerves and are usually found in head, neck, mediastinum, and retroperitoneum, rarely in pelvic and genitourinary organs, especially in seminal vesicles.^[4]^ Most cases of schwannoma occur between the ages of 20 and 50 and are sporadic, but 10% are associated with familial syndromes including type-2 neurofibromatosis, Schwannomatosis, and Carney complex.^[5]^

We report an unusual case of solitary seminal vesical schwannoma treated with conservative strategy for 20 months.

## 2 Case presentation

**Figure 1:**
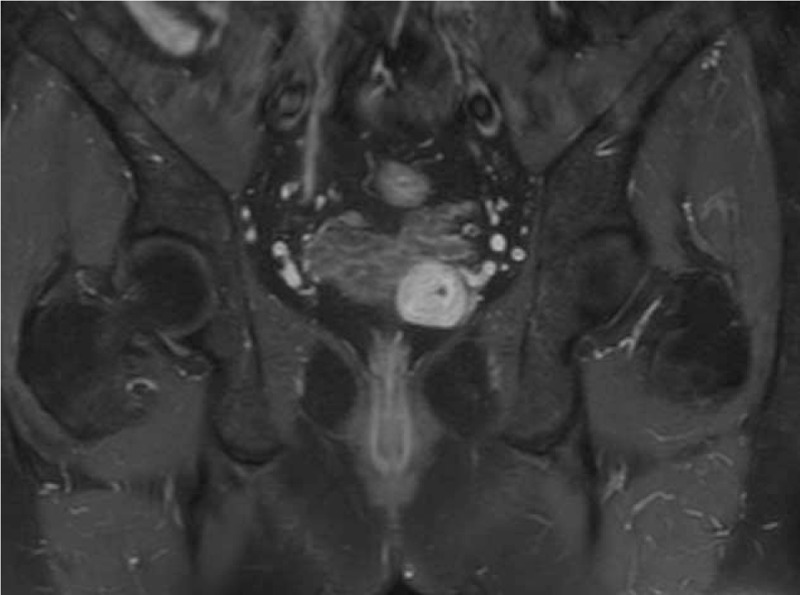
Pelvic MRI showed a solid enhancing mass on the left seminal vesicle on contrast-enhanced T1-weighted image.

**Figure 2:**
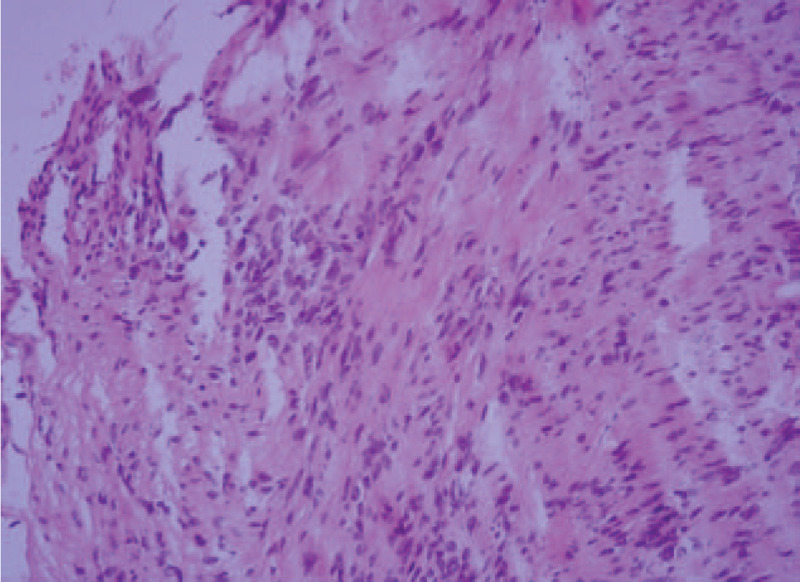
Interlacing fascicles of spindle cells. Hematoxylin-eosin staining, original magnification ×100.

In December 2016, as a re-examination after ureteroscopic lithotripsy, a 42-year-old man underwent an abdominal computed tomography (CT) scan, which showed a well-defined solid mass, measuring 3.2 cm × 3.0 cm, localized in the left seminal vesicle. He denied any weight loss nor other constitutional symptoms and had no positive family history or other medical history, except receiving ureteroscopic lithotripsy 1 month ago, due to the right ureteral calculi. On physical examination, digital rectal examination confirmed a palpable mass in the left seminal vesicle. The serum levels of prostate specific antigen and carcinoembryonic antigen were 0.860 μg/L (normal range: 0.00–4.00 μg/L) and 1.1 μg/L (normal range: 0.00–5.00 μg/L), respectively. Magnetic resonance imaging (MRI) of the pelvis indicated a mass in the left seminal vesicle, measuring 2.7 cm × 2.9 cm, manifested as isointense on T1-weighted images, hyperintense on T2-weighted images and severe enhancement on contrast-enhanced T1-weighted images (Fig. 1), with no evidence of local extension of the tumor to the surrounding tissues as well. Transrectal ultrasound-guided biopsy was carried out and pathological examination of the specimen revealed a schwannoma of the left seminal vesicle (Fig. 2), strongly positive for S-100, and negative for EMA, CD34, SMA, and desmin.

Conservative treatment, comprised of periodic CT or MRI, was instituted after a discussion with the patient, who was concerned with the potential impotence after surgery. In August 2018, the patient underwent pelvic CT, which demonstrated the size of the lesion was approximately similar to that in December 2016. No significant genitourinary symptoms had been reported during the follow-up.

## 3 Discussion

Schwannomas of the seminal vesicle are usually asymptomatic until they reach a significant size and cause symptoms then, either from a mass effect or by nerve compression. On digital rectal examination, an enlarged seminal vesicle is usually not palpable. The area above the prostate, however, can be enlarged and compressible if the seminal vesicle is dilated or firm and indurated if the organ contains tumor. These findings indicate further investigation. CT and MRI can provide similar information in evaluation of size, location, extent, and distant spread of the tumor. Transrectal ultrasound-guided biopsy may be helpful for preoperative diagnosis only if the sample contains enough Schwann cells to visualize microscopically, but it is not valid in cystic cases, because biopsy probably aspirates hypocellular fluid instead of tissue from the surrounding cellular areas.^[6]^

Morphologic and immunohistochemical features help distinguish the schwannomas from malignant peripheral nerve sheath tumors. Both grossly and microscopically schwannomas commonly contain areas of hemorrhage and cystic degeneration. Within these tumors two distinct patterns of cell growth may be differentiated histologically: Antoni A and Antoni B. Verocay bodies (hypercellular areas containing spindle shaped cells with palisaded nuclei) are characteristic features of the former while the latter has loose, haphazard arrangements of cells in myxomatous stroma. Further characteristics of Antoni B are large vascular spaces with hyalinized walls. The presence of S-100 protein on immunohistochemical staining is also common with Schwann cells.^[7]^ The presence of Schwannian whorls, a peritumoral capsule, subcapsular lymphocytes, macrophage-rich infiltrates, and the absence of fascicles favors the diagnosis of cellular schwannoma, while the presence of perivascular hypercellularity, tumor herniation into vascular lumens, and necrosis favors malignant peripheral nerve sheath tumor. Moreover, Ki-67 labeling indice ≥20% is highly predictive of malignant peripheral nerve sheath tumor.^[8]^

When it comes to the alternative of conservative strategy or aggressive surgical therapy, age, preference, general health of the patient, and symptoms should be taken into account. Currently, the mainstay of curative treatment remains surgical excision, which nevertheless may induce surgical complications, including sexual dysfunction, ureteral, rectal, and cystic injury. The deep location of the seminal vesicles makes it difficult to reach them. Different surgical approaches, such as transperineal, para/retrovesical, transvesical, and laparoscopic have been described and there‘s no consensual first choice between them. Typically transvesical access requires an incision of bladder to expose the seminal vesicles, while transperineal access demands dissection of rectum off the posterior prostate and may injure neurovascular bundle as well. On the contrary, the laparoscopic approach follows the method of retrovesical open surgery but provides straightforward access and magnified view with minimal postoperative morbidity, and the seminal vesicles can be dissected free from the bladder and the prostate without entering bladder or rectum.^[9]^ Therefore, we concluded that laparoscopy can offer some advantages over traditional open surgery in resection of seminal vesical schwannomas. In 2017, the first documented complete excision of a seminal vesical schwannoma utilizing a robotic assisted laparoscopic approach was reported.^[10]^ Additionally, in schwannomas of other organs, local recurrence has been reported and associated with incomplete excision, with the rate ranging from 16% to 54%.^[3]^

Regular follow-up examination is essential for conservative strategy, as it is in our case. If any significant symptom or progress in size occurs, surgical interventions will be performed as planned. Long-term follow-up of schwannomas in other organs can guide us in deciding a conservative strategy. Suryanarayanan et al studied 436 patients with vestibular schwannoma, managed conservatively for a median of 3.3 years. Only one-third of vestibular schwannomas grew, all of which were identified within the first 4 years of follow-up. In the sporadic group, the tumor growth rate correlated positively with the initial tumor size, that was tumors of 15 mm diameter or more had a significant higher probability of growth.^[11]^ Hajioff et al studied 72 patients with vestibular schwannoma, treated conservatively for a median of 121 months. Active intervention was eventually performed on 25 patients, 75% of whom failed conservative management in the first 5 years of follow-up and the clinic outcomes of whom were not different from those who underwent initial primary treatment.^[12]^ Exceedingly rare cases were reported to undergo malignant transformation and of poor prognosis.^[13]^

## 4 Conclusion

Schwannomas of the seminal vesicle are rare and mostly benign neoplasms that may produce significant symptoms requiring surgery. Further study is necessary to determine the optimal therapy method in asymptomatic cases, of which patient preference and tumor size should be taken into consideration. In our case, conservative treatment seems effective, and the patient would be followed up for a period of at least 5 years, owing to the large size of the tumor and relevant probability of growth.

## Acknowledgments

We thank Dr Sinian Zheng for taking care of the patient.

## Author contributions

**Conceptualization:** Peng Zhang, Ming Yang.

**Investigation:** Peng Zhang, Ming Yang.

**Writing – original draft:** Peng Zhang, Ming Yang.

**Writing – review & editing:** Peng Zhang, Ming Yang.

## Abbreviations

Abbreviations: CT = computed tomography, MRI = magnetic resonance imaging.
